# The immunogenicity of midbrain dopaminergic neurons and the implications for neural grafting trials in Parkinson’s disease

**DOI:** 10.1042/NS20200083

**Published:** 2021-09-13

**Authors:** Shamma Qarin, Sarah K. Howlett, Joanne L. Jones, Roger A. Barker

**Affiliations:** 1 Cambridge Centre for Brain Repair, Forvie Site, Robinson Way, Cambridge, UK; 2 Wellcome-MRC Cambridge Stem Cell Institute, Cambridge Biomedical Campus, University of Cambridge, Puddicombe Way, Cambridge, UK; 3 Department of Clinical Neurosciences, Clifford Allbutt Building, Cambridge Biomedical Campus, University of Cambridge, Hills Road, Cambridge, UK

**Keywords:** dopaminergic, human foetal ventral midbrain, immune rejection, immunogenicity, Parkinson’s disease, transplant

## Abstract

Dopaminergic (DA) cell replacement therapies are a promising experimental treatment for Parkinson’s disease (PD) and a number of different types of DA cell-based therapies have already been trialled in patients. To date, the most successful have been allotransplants of foetal ventral midbrain but even then, the results have been inconsistent. This coupled to the ethical and logistical problems with using this tissue has meant that an alternative cell source has been sought of which human pluripotent stem cells (hPSCs) sources have proven very attractive. Robust protocols for making mesencephalic DA (mesDA) progenitor cells from hPSCs now exist and the first in-human clinical trials have or are about to start. However, while their safety and efficacy are well understood, relatively little is known about their immunogenicity and in this review, we briefly summarise this with reference mainly to the limited literature on human foetal DA cells.

## Introduction

The progressive degeneration of the A9 dopaminergic (DA) neurons in the substantia nigra pars compacta of the ventral midbrain lies at the heart of the pathology of Parkinson’s disease (PD) – the second most common neurodegenerative disorder, affecting approximately 1% of the population aged over 65 years [1,2]. Such degeneration results in some of the core motor features of this condition including bradykinesia and rigidity as well as certain cognitive deficits around executive function. However, the clinical features of PD are not limited to motor problems and all patients have a range of non-motor abnormalities which reflects widespread pathology that is found both within the central nervous system (CNS) and at other sites such as the enteric nervous system. Nevertheless, the critical importance of DA loss in PD is evident by virtue of the fact that L-dopa administration produces marked clinical benefits, especially in patients with early disease. However, this therapy tends to lose efficacy over time, firstly because of progressive DA neuronal loss as the disease evolves, and secondly, because it leads to the development of side effects, most notably L-dopa-induced dyskinesias. Grafting of new DA cells into the brain of patients with PD is one potential means of circumventing these problems.

To date, a number of different types of dopamine cell-based therapies have been considered and even trialled in PD patients [3]. The most successful, so far, have been those undertaken with human foetal ventral midbrain tissue (hfVM) [4–13] which is dissected from foetuses aged approximately 8 weeks post-conception, collected at the time of termination of pregnancies under strict ethical regulations. Despite the success of this approach in pre-clinical animal models, hfVM transplants have yielded inconsistent results in human trials in terms of graft survival and efficacy as well as generating their own side effects including graft-induced dyskinesias (GIDs; [3]). This coupled to the significant logistical and ethical issues associated with the use of such tissue [14], has meant that other cell sources have been sought, of which human pluripotent stem cell (hPSC)-derived mesencephalic DA (mesDA) progenitor cells hold great promise as an alternative [15].

Robust protocols now exist that allow for human embryonic stem (ES) cells or human-induced pluripotent stem (iPS) cells to be differentiated consistently into committed mesDA neuroblasts *in vitro* [16–19]. These *in-vitro* differentiated mesDA progenitors have also been proven to be effective in pre-clinical animal models of PD in terms of graft survival, DA release, local innervation and reversal of behavioural deficits [16–18]. However, their safety and efficacy in patients is unknown but early trials with these types of grafts are now starting [20,21].

The use of therapies of this type brings with it a number of challenges including those linked to the controlled differentiation of the cells to authentic A9 DA neurons without cell overgrowth/tumour formation, graft survival and connectivity, functional efficacy and finally their immunogenicity and the need for host immunosuppression [22]. This review focuses on this last issue, namely their immunogenicity.

## The immune system and the brain

**Figure 1:**
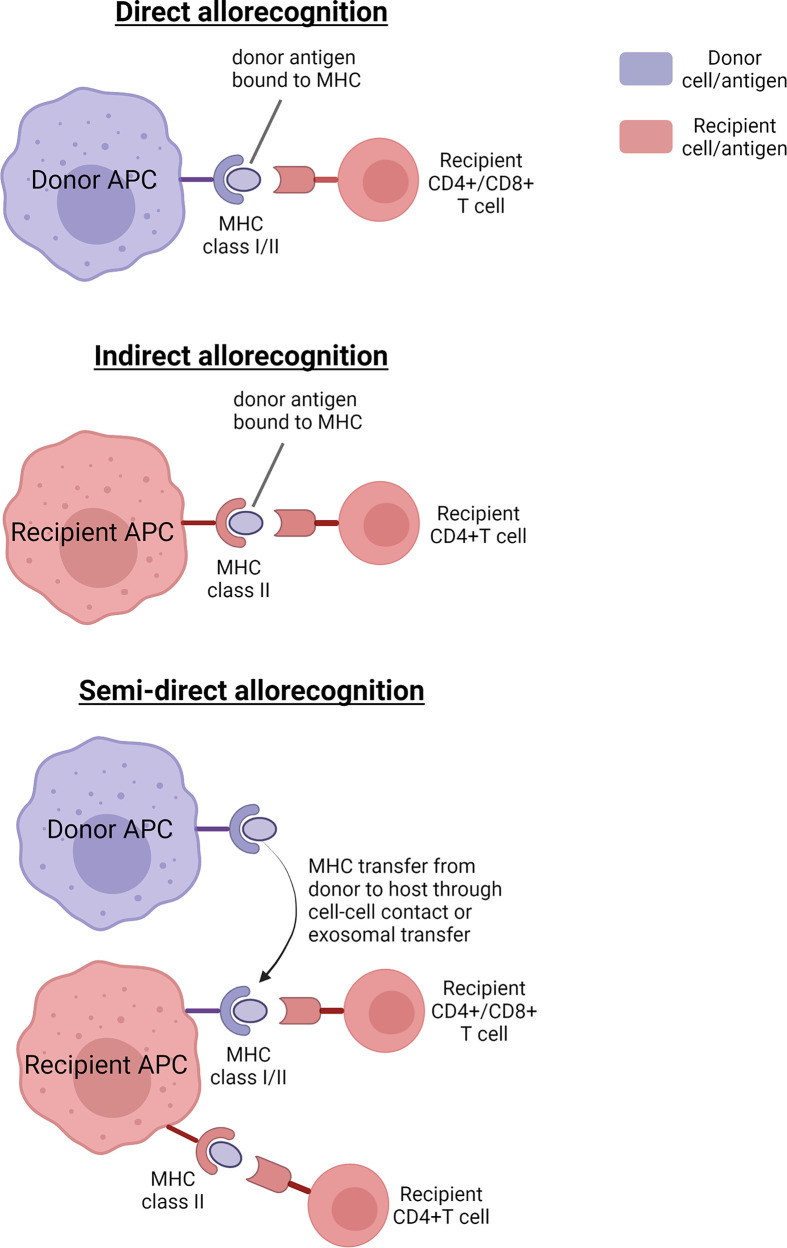
Types of alloantigen recognition by T cells

Immunogenicity of a transplant is defined as the ability of the cellular antigens in the grafted cells or tissues to evoke an immune response in the host leading to an infiltration of lymphocytes into the graft site and potential rejection. Such responses are mainly of two types – cellular and humoral immune responses. The cellular immune response is mainly driven by T cells which recognise foreign alloantigens presented with major histocompatibility complex (MHC) on the cell surfaces of antigen-presenting cells (APCs); co-stimulatory molecules also play a role in this interaction. This alloantigen recognition by T cells can be through one of three types – direct, semidirect and indirect (see Figure 1).

In direct allorecognition, alloantigens from the graft are presented by donor APCs to T cells of the host, whereas indirect allorecognition is one in which donor-derived antigens are captured by host APCs and then presented to host T cells eventually eliciting an immune response. In semidirect recognition, cell-to-cell contact between donor and recipient APCs may transfer intact donor MHC molecules to recipient APCs, or donor APCs may release exosomes containing MHC molecules which then fuse with the recipient APC membrane. As a result, recipient APC becomes chimeric with both donor and recipient MHC molecules, which can then stimulate both direct and indirect pathways [23,24].

The humoral immune response is primarily driven by B cells whereby, upon recognition of foreign antigens from the graft, they differentiate to plasma cells and produce antibodies against the antigens to drive antibody-mediated rejection. In addition, the transplant may also express epitopes to which the host has pre-formed antibodies – e.g., as is seen in porcine xenografts with host anti-Gal antibodies [25]. This B-cell response is considered a major barrier in transplantation because conventional immunosuppressants largely target T cells. Such humoral responses and the generation of antibodies can activate the complement cascade which facilitates antibody-mediated rejection which is hyperacute for xenogeneic tissue placed in the periphery but much slower with allografts. Complement additionally recognises apoptotic and necrotic cells and modifies antigen presentation to B and T cells [26].

However, allogeneic graft rejection of cell suspension transplants in the CNS is different from that seen with peripheral organ transplants. The first difference relates to the use of cell suspensions in the brain as opposed to whole organs – as the latter have a vasculature with a donor-derived endothelium which is highly immunogenic. Secondly, foetal neural cells (which have been the main cells used for grafting into the brains of patients with PD) have been shown to express no or only low levels of immunogenic molecules [27,28]. The third major difference relates to the blood–brain barrier (BBB) that is jointly maintained by endothelial tight junctions, endothelial basal lamina and astrocyte endfeet processes. This BBB makes the brain a relatively immune privileged site given it represents a physical barrier to molecules of high molecular weight and cell transmigration from the circulation into the brain. Fourthly, the CNS lacks professional APCs. Microglia, which are the resident CNS macrophages, unlike peripheral dendritic cells (DCs), have reduced capacity in presenting antigens to induce T-cell proliferation. Fifthly, the CNS has a poorly developed lymphatic system (although of late, this has been revisited through the discovery of the glymphatic system ([29]; see below) and finally, reactive astrocytes can be anti-inflammatory and immunosuppressive [30] which could prolong graft survival in the brain.

However, this immune privilege status of the brain is not absolute and permanent in that it normally allows access of some peripheral T lymphocytes into the brain. Microglia have also been shown to attract peripheral DCs into the inflamed brain [31] as will be the situation immediately following cell implantation. In addition, pericytes and perivascular macrophages found in the Virchow–Robin spaces are in continuous migration across the cerebrovascular endothelium [32] and so can be involved in graft recognition and rejection. Finally, the newly discovered glymphatic system means that centrally placed antigens have relatively easy access to the peripheral immune system. This system consists of a network of perivascular channels and clears CNS waste – neurotoxic protein aggregates and metabolites, mostly during sleep with the help of the cerebrospinal fluid (CSF) – into the subarachnoid space or cervical lymph nodes to join the main lymphatic system 33]. CSF also drains into the cervical lymphatic system through the cribriform plate and through cranial and olfactory nerve sheaths [34,35]. This means that antigens shed from an intracerebrally placed graft will enter the main lymphatic drainage, where they will encounter naïve T cells, potentially triggering a peripheral immune response which could direct activated lymphocytes to the graft site. Taken together, the brain can be regarded as a site where immune responses can occur, however in an adapted form, especially as neural grafting will disrupt the BBB and induce a local trauma-induced inflammatory response.

Allogeneic rejection could also be specifically different in the aged PD brain. CSF production declines with age meaning there is a reduction in glymphatic activity [36]. In addition, the rapid transport of CSF depends on aquaporin 4 (AQP4) channels which have been found to be mislocalised with age in mouse brains [37,38] and this coupled to age-related arterial stiffening and reduced arterial pulsatility could all further adversely impact on glymphatic clearance [37,39]. In addition, sleep disturbances [40] can also contribute to a glymphatic decline and sleep is commonly disrupted in PD [41], which may also contribute to alterations in glymphatic function. Indeed, a recent study [42] with idiopathic PD patients reported that there is a significant decline in meningeal lymphatic drainage as well as a significant delay in deep cervical lymph node perfusion. Interestingly, in the present study they showed that injection of pre-formed α-synuclein fibrils in mouse brains resulted in delayed glymphatic drainage and increased meningeal inflammation which in turn aggravated α-synuclein pathology and triggered motor dysfunction. However, whilst this decline could be detrimental for the disease process, it may also be beneficial for graft survival as the reduced flow of graft antigens into the lymphatic system could possibly promote graft survival. However, this needs further exploration.

## The immunogenicity of human foetal dopamine cells *in vitro*

*In vitro* experiments looking at the immunogenicity of DA neurons are limited. One of the first studies conducted by Goya et al. [27] on the immunogenicity of hfVM neural progenitor cells (NPCs) *in vitro* reported an initial undetectable expression of MHC I or II molecules. However, their expression was up-regulated by pro-inflammatory cytokines. This indicated that in an inflammatory environment, such as would be the case at a CNS graft site immediately post-operatively, they could be up-regulated potentially making the transplanted cells more susceptible to immune rejection. In co-cultures with peripheral blood mononuclear cells (PBMCs) *in vitro*, human NPCs elicited T-cell proliferation, whilst culturing them with purified allogenic T cells elicited a much weaker or undetectable response. This indicated that foetal DA cells were not capable of stimulating T cells directly, perhaps because they did not express co-stimulatory molecules, and so the response was largely driven by indirect allorecognition through host APCs. Overall, these data indicated that hfVM NPCs were weakly immunogenic *in vitro*.

These studies have been extended by other groups to involve other relevant cells including NPCs from other brain regions, such as the foetal forebrain. These studies have also shown that the cells are only mildly immunogenic with limited MHC expression and no significant T-cell proliferation [28,43,44]. In contrast, other studies came to different conclusions. Liu et al. [45], using human embryonic stem cell (hESC)-derived and foetal spinal cord-derived NPCs, showed that PBMC proliferation could be provoked. Similarly it was found by Huang et al. [46] that there was PBMC proliferation when these cells were challenged by allogeneic human induced pluripotent stem cell (hiPSC)-derived NPCs in co-culture but not when autologous hiPSC-derived NPCs were used. Despite similar experimental methods and time allowed in co-culture, the variable findings raise questions about heterogeneity in expression of immune molecules and the subsequent immunogenicity of NPCs derived in different ways and/or from different brain regions [27].

One relevant factor that might be important to this T-cell response is the expression of co-stimulatory molecules on the grafted cells. Co-stimulatory molecules, which are important in the interaction between APCs and T cells and orchestrating an immune response, are absent from DA progenitors [27] and also absent [43,47] or expressed at very low levels [28,45] from NPCs from other CNS regions, such as those derived from the forebrain and spinal cord. These are not up-regulated even in an inflammatory environment suggesting that such cells lack an important prerequisite for proper antigen presentation and recognition.

One interesting factor that could further contribute to the low immunogenicity of neural progenitors *in vitro* is that they may actually be slightly immunosuppressive [45,48]. NPCs, in general, produce a number of factors such as, TGF-β, nitric oxide, prostaglandin E2, and haem oxygenase which might have an effect on the host immune response. It has been demonstrated in some studies [44,45] that TGF-β secreted by neural cells can inhibit the proliferation of T cells. A second factor could be haem oxygenase-1 [49], which has been shown to be secreted by foetal rat NPCs and found to suppress T-cell proliferation, the function of which was counteracted by haem oxygenase inhibitors. Two other possible factors that may contribute to the anti-inflammatory activity of NPCs *in vitro* includes nitric oxide and prostaglandin E2 [50]. Activated T cells induced murine neural stem cells in co-culture to produce these two compounds which in turn inhibited T-cell proliferation.

To summarise, most of the *in vitro* studies have suggested that neural progenitors, regardless of their site of origin, have low intrinsic immunogenicity. As to whether they can trigger a T-cell response *in vitro* varies from study to study but at best the response is modest and, in some cases, it has even been shown that NPCs are themselves slightly immunosuppressive. Thus, the extent to which transplants of such cells will require immunosuppression of the host remains unclear.

## The immunogenicity of foetal dopamine cells *in vivo* in pre-clinical animal models

The immunogenicity of DA cells *in vivo* in animal models of PD has been studied immunohistochemically. Early studies [47] with mouse foetal DA tissue allografted into adult mouse brains showed no infiltration of immune cells, at the time of killing, 6–7 weeks after grafting, regardless of the donor–host histocompatibility. The DA grafts also survived longer – up to 15 weeks, compared with similarly derived skin allografts. However, host immunisation with a prior graft was observed to be similar whether the prior graft was neural or skin. Together, these results suggest that neural cells are immunogenic when grafted into the adult CNS, but that the immune response to them might be delayed. This could be explained by delayed drainage of grafted antigens to the peripheral immune cells and/or by the fact that as the graft matures within the host, its growth and reinnervation may be accompanied by increased expression of immunogenic molecules, including MHC. This could make the graft more immunogenic over time, hence more susceptible to rejection [51]. Also, chronic inflammation seen following the trauma of intracerebral grafting of tissue could drive up MHC expression in the graft itself and this could also eventually lead to rejection. Such delays would be unfavourable for both the survival and function of DA grafts leading to behavioural deterioration which may only become apparent after withdrawal of immunosuppression, even when this is done some time after the original tissue was implanted [52].

It has been demonstrated that MHC I expression in allografted cells is low in-line with what has been seen in the *in vitro* studies discussed (see above). However, on transplanting allogeneic foetal tissue in rats, immune cells of microglia-like morphology [53] as well as T cells could be detected around the grafts [52,54] as has been seen in some PD patients in receipt of allografted hfVM tissue [6,8,11] – all of which suggest that the graft may induce a low-grade rejection/immune response from the host. In-line with this, another study sought to investigate the immunogenicity of mouse-derived foetal cells in autologous versus allogeneic grafts. In the absence of immunosuppression, even syngeneic and allogeneic grafts elicited a host immune response [55] indicating that these cells are immunogenic. The possible significance of all of this was further demonstrated in another study [56] which was designed to understand whether DA allografts were rejected upon immunisation of the host with a prior graft. In the present study it was reported that, all of the second neural allografts were rejected – namely the host had been sensitised to the tissue immunologically by the first allograft. Thus, reducing prior graft antigenic presentation by undertaking single bilateral transplants in patients and using immunosuppressive agents for at least 1–2 years might be needed to reduce such an event happening in any trial in patients, although anecdotally in studies that have done serial hfVM grafts in PD patients, this has not been seen to be a major issue [57].

Of interest in this respect is a very recent study [20] that looked at the immunogenicity of *in vitro* differentiated mesDA progenitors from human ES and patient-derived iPS cells post-intrastriatal grafting in NOD-SCID-γ IL2 knockout (NSGs) humanised mouse models. They reported an absence of immunogenicity for autologous mesDA cells grafted in the patient-humanised mouse models. However, allogeneic hESC-derived mesDA progenitors were highly immunogenic, as indicated by a high infiltration of CD4^+^ T cells into the graft site accompanied with neural cell death. Additionally, both hESC and patient iPSC-derived cells were rejected in allogeneic humanised mouse models. In contrast, another study [58] using monkey foetal DA cells allografted into monkey brains, found no evidence of an immune response. However, more recently Morizane et al. [59] compared autologous and allogeneic non-human primate DA grafts and showed that monkey iPSC-derived autologous DA cells grafted into monkeys evoked only a negligible immune response compared with allografts which exhibited considerable microglial activation and leucocyte infiltration. However, this response could be abrogated through using a single immunosuppressive agent tacrolimus (FK506), a calcineurin inhibitor [60].

Other neural cells seem to behave similarly to DA neurons. Foetal NPCs, in general, have been described as having low immunogenicity as they have been shown to survive in non-immunoprivileged sites without any host immune reaction [61]. In this study, neural cells from neonatal mouse forebrains (which had undetectable MHC I and II expression levels) were allografted under the kidney capsule of histoincompatible mice and the grafts survived for 4 weeks. However, this is a relatively short time and may explain why they found a difference from that seen in earlier studies by Mason et al. [62] in which foetal neural tissue allografted under the kidney capsule and in immunologically primed rodent hosts was heavily infiltrated with leucocytes and eventually rejected over a period of 10 and 20 days, respectively.

The issue of histocompatibility is an important one as *in vivo* studies indicate that the more incompatibility there is between the MHC of the host and the donor, the greater the rejection response of the neural tissue [47]. In non-human primates, MHC matching of iPSC-derived neurons improved engraftment whereas MHC mismatch caused neuroinflammation [60,63]. However, MHC matching may still fail to prevent long-term rejection even in the brain because of differences in the expression of minor histocompatibility complex (miHC) antigens – endogenous polymorphic peptides which are presented within the variable regions of the MHC I and II molecules. Although the miHC molecules might not be as immunogenic as the MHC molecules, and so, less critical in graft rejection, evidence suggests that the immunogenicity of grafted cells also depends on miHC, and incompatibility in such antigens can be enough to cause rejection [60,64] despite MHC match [65]. In fact, multiple miHC incompatibilities alone may be more immunogenic than MHC differences [66]. MiHC mismatch is susceptible to innate immunity through natural killer (NK) cells and serum complement and in cases where there is donor–host incompatibility in both MHC and miHC molecules, the graft is more likely to induce an acute inflammatory, as well as a long-term rejection response [53,54].

Overall, most of the pre-clinical *in vivo* studies undertaken have reported survival of DA grafts in the allografted situation albeit with a degree of inflammation around and within the graft. However, many of these studies have used relatively short survival times so may miss a delayed chronic rejection response although reassuringly most studies have shown that immunosuppressive drugs, in general, promote allograft survival. As such, these agents would seem to be of value in clinical trials in patients in receipt of allografted neural tissue [44].

## The immunogenicity of human foetal VM allografts in human trials

Human intrastriatal transplants of foetal DA neurons into PD patients were first performed in the late 1980s [9] and to date, most of the work looking at their immunogenicity has relied on looking at the infiltration of immune cells into the graft at autopsy. Human trials with foetal cells have yielded inconsistent results in that initially they showed some efficacy in pre-clinical and open label trials, however, subsequent double-blinded, placebo-controlled trials did not support such conclusions [5,12]. In both trials, some patients showed a significant increase in F-Dopa uptake at the graft site with some motor improvements, but many did not and some even developed complications especially the development of GIDs. The extent to which this is related to any immune rejection of the tissue is debated [67] as these trials took very different approaches to the use of immunosuppressants. Freed et al. [5] used no immunosuppression at all and observed the presence of T cells and MHC-II+ cells in the graft areas up to a year post-surgery. Olanow et al. [12] showed similar results with the presence of activated microglia in the graft areas post-mortem despite robust DA neuron survival, but this was some time after the patient had finished their 6 months of cyclosporin A monotherapy. Interestingly in this trial, there was also a clear clinical deterioration in their grafted patients upon discontinuing of the cyclosporin at 6 months post-surgery, suggesting that there may have been a rejection response that was triggered at this time of withdrawal of these immune suppressing agents which compromised the long-term functionality of the grafts. This suggests that a more prolonged period of immunosuppression might be needed for long-term graft survival.

Other post-mortem studies have also demonstrated the presence of significant numbers of immune cells including microglia, macrophages, T cells and B cells around the transplant without overt evidence of graft rejection [6]. This further implies that seeing an immune response around a graft does not equal graft rejection but that grafted human fVM tissue can induce a low-grade chronic inflammatory response.

In contrast with the above, Mendez et al. [11] showed that foetal DA grafts in the striatum and substantia nigra of PD patients had minimal microglial infiltration into the graft site at post-mortem, 3–4 years later. These patients also received 6 months of immunosuppression (cyclosporin A) which meant that the grafts had survived well without immunosuppression for 3 years. Two more studies [7,13] reported similar results with clinical benefits and no observable immune rejection response at post-mortem with the same immunosuppressive regime. In another long-term study [8], a patient that had been grafted with hFVM had been in receipt of 5 years of immunosuppression (prednisolone, azathioprine and cyclosporine), but was found to have a healthy graft with no inflammation around it, 19 years later when he died.

Taken together, all these data suggest that hfVM allografts can survive long term in the absence of long-term immunosuppression in the PD brain but that there is a critical period when the cells in the graft can induce some form of host immune response. A response that may significantly compromise the integrity and function of the transplant. All of which suggests that a period of immunosuppression post-grafting is probably needed, although the extent and length of that is debated. The extent to which this could be reduced by better matching of the donor cells to the host is also unknown as tissue typing of the host and donor foetuses in these studies was not possible given the number of foetuses needed to graft a single PD patient. However, this will be different with hPSC-derived DA cells.

## Other factors contributing to neural graft immunogenicity and transplant outcome

There are a number of other factors that could alter how the immune system sees a neural graft. First of all, graft site is important [68], as cells placed into the brain parenchyma seem to do better than similar grafts placed into the ventricle. For example, histoincompatible grafts which did not survive in the ventricle did so when placed intraparenchymally [66,69]. In addition, the grafting procedure itself may be an important additional factor. Tissue damage or bleeding at the graft site could also act as a stimulating factor for the host immune system to respond given that macrophages normally accumulate at the site and present the dead donor cell material to host immune cells [70] and thus the graft could be compromised in a bystander way. Linked to this is the fact that the surgical procedures needed to implant the cells will also influence the immune response – surgical trauma will disrupt the BBB so allowing circulating immune cells access to the graft.

Tissue typing may also be another critical factor as Lopez-Lozano et al. [71] assessed ABO typing between fVM tissue and recipient and observed that ABO-matched grafts survived while the mismatched grafts did not. Thus, tissue typing of donor and host is desirable which is, however, only feasible with stem cell sources [60].

Preparation of tissue could also contribute to the immunogenicity and eventual immune reaction from the host. Solid tissue allografts are likely to be more immunogenic in that they contain donor-derived blood vessel endothelium, expressing high levels of donor MHC-I antigens, which might have a greater capacity for generating a delayed inflammatory response [72]. This could also be one of the factors accounting for the immune response that was observed in the two NIH-funded trials [5,12] since the grafted tissues were implanted as tissue ‘noodles’ and solid pieces, respectively. In contrast, Mendez et al. [11] used cell suspension grafts and observed only little microglial activation – a situation that is more analogous to that which will be seen with hPSC-derived DA cells given these will be delivered to the CNS as a cell suspension.

The use of L-Dopa treatment after transplantation could also conceivably influence the immune response to the graft. Breger et al. [73] found that, although L-Dopa treatment did not affect mouse xenograft survival in rats, it significantly promoted immune responses to the grafts (indicated by high infiltration of microglia and lymphocytes). The mechanism underlying this phenomenon is far from clear and the authors of this work [73] hypothesised that it could be due to L-Dopa; (i) interfering with the action of immunosuppressive drugs; (ii) activating or leading to proliferation of immune cells or (iii) L-Dopa impairing the BBB allowing entry of immune cells into the brain. Although only a single study, further work is required to understand the effects of L-Dopa on the immune system, especially given that PD patients in trials of cell therapies are likely to be receiving L-dopa.

Finally, how disease state alters the immune system of the host systemically and locally within the brain at the site of grafting also needs to be better understood. It is well known that in PD, changes in the peripheral immune system have been found [74,75] and this could impact on graft survival. In the first instance, PBMCs from PD patients produce more inflammatory cytokines compared with healthy controls which could adversely affect the grafted cells [76]. Secondly, PD patients have been found to have pathologically hyperactive monocytes [77] as well as T-regulatory cells with an impaired ability to suppress effector T-cell function [78] – which again could potentially allow recruitment of more T cells to the graft site compromising graft survival. The extent to which any PD relevant pathology in the striatum at the site of grafting impacts on graft survival and its immunogenicity is completely unknown.

## Discussion and further research

Overall few studies have looked at the immunogenicity of foetal midbrain DA cells. With regard to *in vitro* work, except for one study with foetal VM cells [27], whatever knowledge we have on their *in vitro* immunogenicity relates more to neural cells derived from various brain regions and not specifically mesDA cells. These data have indicated that neural cells display minimal, but still a degree of, immunogenicity and this has been borne out *in vivo* as well as in human trials in PD patients. In addition to the low innate immunogenicity of foetal neural tissue, it is also clear that the brain is a relatively immune privileged site although aspects of this are being redefined including in the context of age-related CNS diseases.

Despite our limited knowledge and understanding on the immunogenicity of PSC-derived DA neurons, clinical trials have already started with these cells. To date it has been assumed that the immunogenicity of hPSC-derived DA cells is comparable with primary hfVM cells. This is based on the fact that they appear to have a low expression of MHC and co-stimulatory molecules and can be maintained in pre-clinical studies using the same immunosuppressive regimes as though using for hfVM tissue. In addition, it has been noted by many groups that neural stem cells (which are an intermediate stage from stem cells to dopamine neurons) have an immunosuppressive effect *in vitro* (see above).

However, slight differences could arise and in particular, human fVM may be more immunogenic by virtue of containing a greater mix of cell types compared with hPSC-derived DA neuronal products. Human fVM tissue will contain glial precursors that give rise to astrocytes and oligodendrocytes that might also trigger the host immune response given that they drive microglial activation. Tissue damage during dissection of the foetal midbrain sample could also trigger the release of inflammatory cytokines, such as IL-6 and TNF-α, from the surrounding foetal astrocytes and microglia that could contribute to any rejection response [79].

The knowledge we have to date on the immunogenicity of hfVM has therefore helped in the design of these first in-human trials with PSC-derived DA cells. In general, autologous cells – whether DA or NPCs of other brain regions – evoke no or only negligible T-cell response and so will not require any immunosuppressive therapy. However, allogeneic cells will likely be immunogenic to some extent and the greater the degree of histoincompatibility, the greater this rejection response is likely to be. Thus, whilst autologous iPSC-derived DA cells seem to be the most obvious option, this is not currently possible because of the cost of preparing such a product for every patient outside of any concerns about disease susceptibility in the dopamine cells generated. Thus MHC- and miHC-matched allogeneic hPSC-derived DA cells coupled with a limited period of immunosuppression might be the best option for the time being – although even this is not straightforward as banks of cells will be needed for this which cover the common haplotypes of the treated population [28].

One alternative, of course, to all this and to reduce rejection, or even obviate the need to worry about it at all, is through genetically engineering the grafted cells so that they have a knockout of HLA-I/II proteins accompanied by an increased expression of HLA-G or E [80,81]. This increased expression of HLA-G or E could be useful to inhibit NK cell rejection of the grafted HLA-I/II knockout cells because they interact with the killer inhibition receptors of NK cells, hence preventing the rejection process mediated by these cells [82]. However, these will need to be explored further. In this regard, some studies have demonstrated that co-stimulation blockade therapy, such as with anti-CD40L, could mitigate the immune response and enhance long-term graft survival [83,84]. The usefulness of this is however not known in humans.

In summary, we have only a very limited understanding of the immunogenicity of human midbrain dopamine cells and thus more extensive studies are needed to better understand their immunogenicity and its implications for the programmes seeking to take such cells into patients with PD.

## Open Access

Open access for this article was enabled by the participation of University of Cambridge in an all-inclusive *Read & Publish* pilot with Portland Press and the Biochemical Society under a transformative agreement with JISC.

## Abbreviations

APC: antigen-presenting cell
BBB: blood–brain barrier
CNS: central nervous system
CSF: cerebrospinal fluid
DA: dopaminergic
DC: dendritic cell
GID: graft-induced dyskinesia
hESC: human embryonic stem cell
hfVM: human foetal ventral midbrain
hiPSC: human induced pluripotent stem cell
hPSC: human pluripotent stem cell
iPS: induced pluripotent stem
mesDA: mesencephalic dopaminergic
MHC: major histocompatibility complex
miHC: minor histocompatibility complex
NK: natural killer
NOD-SCID: Nonobese diabetic/severe combined immunodeficiency
NPC: neural progenitor cell
PBMC: peripheral blood mononuclear cell
PD: Parkinson’s disease
